# Health Care Providers' Knowledge and Practice Gap towards Joint Zoonotic Disease Surveillance System: Challenges and Opportunities, Gomma District, Southwest Ethiopia

**DOI:** 10.1155/2016/3942672

**Published:** 2016-08-07

**Authors:** Desta Hiko Gemeda, Abiot Girma Sime, Kifle Woldemichael Hajito, Benti Deresa Gelalacha, Wubit Tafese, Tsegaye Tewelde Gebrehiwot

**Affiliations:** ^1^Department of Epidemiology, College of Health Sciences, Jimma University, Jimma, Ethiopia; ^2^School of Veterinary Medicine, Jimma University, Jimma, Ethiopia

## Abstract

*Background.* Health care providers play a crucial role for realization of joint zoonotic diseases surveillance by human and animal health sectors, yet there is limited evidence. Hence, this study aimed to determine knowledge and practice gap of health care providers towards the approach for Rabies and Anthrax in Southwest Ethiopia.* Methods.* A cross-sectional survey was conducted from December 16, 2014, to January 14, 2015. Eligible health care providers were considered for the study. Data were entered in to Epi-data version 3.1 and analyzed using SPSS version 20.* Results.* A total of 323 (92.02%) health care providers participated in the study. Three hundred sixteen (97.8%) of participants reported that both human and animal health sectors can work together for zoonotic diseases while 96.9% of them replied that both sectors can jointly conduct surveillance. One hundred seventeen (36.2%) of them reported that their respective sectors had conducted joint surveillance for zoonotic diseases. Their involvement was, however, limited to joint outbreak response.* Conclusion.* There is good opportunity in health care providers' knowledge even though the practice was unacceptably low and did not address all surveillance components. Therefore, formal joint surveillance structure should be in place for optimal implementation of surveillance.

## 1. Introduction

It is widely recognized that zoonotic diseases control and elimination require a joint approach by animal and human health sectors [1, 2]. Joint surveillance system using One Health approach is the most effective and efficient way of protecting human and animal populations from zoonotic disease like rabies and anthrax in low income countries [3–5]. However, implementation of such surveillance approach in these countries is hampered by several factors: multiple disease challenges, unmotivated One Health workforce, remoteness, lack of appropriate working tools/infrastructure, and low budget, just to mention a few. These factors often led to emergence and spread of zoonotic diseases like rabies and anthrax as these countries are home to those zoonotic diseases [6].

Similar to other developing countries, one sector approach and weak preexisting animal and human health surveillance system is widely practiced in Ethiopia in general and in Jimma Zone in particular. This approach is characterized by delayed outbreak detection and management of two of the most important zoonotic diseases (rabies and anthrax) in both humans and animal population in Jimma. Such case detection is often after massive loss of human and animal lives and probably after occurrence of several outbreaks detected/undetected in both humans and animals population. Apart from delayed outbreak detection and management, the preexisting weak surveillance system in the countries can jeopardize rabies and anthrax control and eradication efforts of the Sub-Saharan African countries [7].

Jimma Zone, Southwest of Ethiopia, is home to rabies and anthrax, the two common zoonotic diseases. Studies showed that on average three victims visit Jimma town antirabies health centre per day for postexposure prophylaxis excluding victims going to traditional healers. Thirteen deaths due to rabies were reported from Jimma health centre between mid-October 2012 and mid-January 2013 [8]. In December 2013, a massive outbreak of rabies has occurred and claimed the life of ten individuals in Shabe Sombo district of Jimma Zone [9]. Likewise, severe outbreak of anthrax in Jimma Zone was detected in March 2014 which led to public panic as cases were detected even in municipal abattoir in cattle and sheep. The municipal abattoir, hotels, and restaurants in the town were closed for about three weeks in April 2014 by local authorities causing high socioeconomic impact to the local community [10].

Joint zoonotic diseases surveillance approach is not yet formally established in low income countries like Ethiopia. However, there are informal joint practices usually limited on outbreak management. For the realization of the joint zoonotic disease surveillance approach, health care providers in human and animal health sectors play a crucial role. However, there is limited evidence on providers' knowledge and practice related opportunities and challenges towards joint surveillance for zoonotic disease. Hence, this study was aimed at determining knowledge and practice gap towards such surveillance approach among health care providers in both sectors of the district.

## 2. Methods and Participants

### 2.1. Study Setting

The research was carried out in Gomma district of Jimma Zone of Oromia Region. Agaro is a capital town of Gomma district located at altitude ranging from 1,380 to 1,680 meters above sea level. However, some points along the Southern and Western boundaries have altitudes ranging from 2,229 to 2,870 meters. The projected total population of the district from the 2007 national census in 2014 is 246, 381 (which is 51,652 households) of which 3,094 (5.99%) were urban households and 48558 (94.01%) rural households [11] residing in 36 rural and 3 urban Kebeles (Kebele is the smallest administrative unit in Ethiopia) [12]. Data were collected from December 16, 2014, to January 14, 2015.

### 2.2. Study Design, Population and Sampling

A community based cross-sectional study design was conducted on all health care providers in Gomma district (351 animal and human health service providers). One hundred thirty health care providers from Agaro health centre, 82 health extension workers from all Kebeles of the district, 9 human health surveillance focal persons from Gomma district level and animal health service providers 1 veterinary doctor, 9 animal health surveillance focal persons, 65 development agents (these are professionals deployed at Kebele level to promote development through primary prevention of disease in animals), and 55 animal health assistants (these are professionals who provide curative care for sick animals at animal clinics) in all Kebeles of the district constitute study population.

### 2.3. Data Collection Procedure

Interviewer administered structured questionnaire was adapted [13] and developed from different sources. The tool was prepared in English and translated to Amharic language. Then, the translated tool was pretested among health care providers in similar settings in another district of the zone and used for data collection. Four trained graduate program students in Veterinary and Public Health Epidemiology from Jimma University collected data on background characteristics, knowledge, and practice gap of service providers on joint zoonotic disease (rabies and anthrax) surveillance system using face-to-face interview. Data collection process was closely supervised by two veterinary and public health epidemiologists.

### 2.4. Data Processing and Analysis

Data were checked, edited, and entered into Epi-data version 3.1. Data were then cleaned for outliers and missing values and analyzed using SPSS version 20. Descriptive analysis was done to generate summary values for variables on background characteristics, knowledge, and practice of service providers.

### 2.5. Ethical Considerations

Ethical clearance was obtained from both Health Sciences College and School of Veterinary Medicine Institutional Review Boards of Jimma University. Permission letter was sought from both human and animal Gomma district health offices to conduct the research. Before data collection, the objective of the study was explained to the participants and data collection was commenced only after obtaining verbal consent. Finally, the data were used for the research purpose only.

## 3. Results

### 3.1. Sociodemographic Characteristics

From a total of 351 human and animal health service providers, 323 (92.02%) participated in the study, among which 91.9% were human health providers. The majority (65.6%) of the respondents were females with median age of 29 years. Similarly, most (52.9%) respondents served for 61 and above months with a median of 72 months. Nearly three-fourths (74.0%) and one in ten (11.5%) of the participants were working in health centres and health posts, respectively. Most of the participants had B.S. degree (56.3%) followed by diploma (36.2%) holders (Table 1).

### 3.2. Knowledge Gap of Health Service Providers on Rabies: Transmission, Reservoir, and Prevention

Almost all (99.7%) respondents heard about rabies; however, 29 (9%) and 3 (0.9%) health care providers replied that rabies do not attack dogs and humans, respectively. Most of the respondents mentioned that rabies can be transmitted from sick animal to human (98.1%) and from sick animal to animal (96.6%). Similarly, more than seven out of ten respondents mentioned that vaccination of animals (73.9%), isolation of sick animals (80.7%), creating community awareness (93.5%), and conducting active surveillance (87.6%) can prevent the acquisition and transmission of rabies in humans and animals (Table 2).

### 3.3. Knowledge Gap of Health Service Providers on Anthrax: Transmission, Reservoir, and Prevention

Almost all (97.5%) respondents heard about anthrax; however, 104 (33%), 109 (34.7%), and 251 (79.7%) of health care providers replied that anthrax do not attack domestic animals, humans, and wild animals, respectively. More than two-thirds (68.3%) of the respondents replied that anthrax can be transmitted from sick animal to human. Similarly, more than half of the respondents mentioned that vaccination of animals (64.1%), isolation of sick animals (62.9%), creating community awareness (65.1%) and conducting active surveillance (55.6%), wearing protective clothing (55.6%), disinfection of contacts/materials (62.2%), and proper burial of dead animal/human (63.2%) can prevent the acquisition and transmission of anthrax in humans and animals (Table 3).

### 3.4. Knowledge Gap of Health Care Providers towards Joint Surveillance for Rabies and Anthrax

More than 96% of the respondents replied that occurrence of both rabies and anthrax in human and animal population is reportable in Ethiopia. The finding revealed that 97.8% of the respondents reported that both human and animal health sectors can work together for zoonotic diseases and similarly 96.9% of them replied that both human and animal sectors can jointly conduct surveillance on zoonotic diseases like rabies and anthrax (Tables 2 and 3). A total of seven human and no animal health care providers were not aware whether the two diseases' occurrence in human population is reportable. Similarly, one animal and twelve human health care providers replied that the occurrence of rabies and anthrax in animal population is not reportable. One animal and nine human health care providers replied that the two sectors cannot undertake joint surveillance for zoonotic diseases like rabies and anthrax.

### 3.5. Practice Gap of Health Care Providers and Their Respective Sectors on Rabies and Anthrax Joint Surveillance

Just over a third (36.2%) of health care providers reported that their respective sectors had conducted joint surveillance for zoonotic diseases of public health importance in the past; however, almost all (99.1%) of the respondents who had joint surveillance experience reported that their involvement was limited to outbreak response. Only less than 11% of the respondents mentioned that they were involved in joint surveillance planning, surveillance data exchange, monitoring, and evaluation of surveillance activities. Seven respondents (2.2%) reported that their sectors have joint surveillance structure for zoonotic disease of which only 5 reported that level of integration between human and animal sectors was between health extension workers and development agents (DAs), health centres and veterinary clinics, and human and animal surveillance focal persons, respectively. Similarly, only six respondents reported that they had joint surveillance manual for zoonotic diseases in their respective health institutions (Table 4). One hundred eighty-six human and twenty animal health care providers reported that they did not conduct joint surveillance on zoonotic disease like rabies and anthrax.

## 4. Discussion

More than nine in ten of the respondents replied that both human and animal health sectors can jointly conduct surveillance on rabies and anthrax. However, only a third of health care providers reported that their respective sectors had conducted joint surveillance for zoonotic diseases of public health importance in the past one year preceding the survey and their involvement was limited to joint outbreak response. The respondents also mentioned that there is formal joint surveillance structure even during outbreak response in the district.

Almost all (99.7%) respondents heard about rabies and this was higher than (45.8%) what was reported by Tanzanian animal health workers [14]. The variation could be explained by the fact that study participants were only animal health care providers in Tanzania unlike the current study. In addition, in Tanzania the study was conducted from 2001 to 2002, during which public health importance of zoonotic diseases was poorly given due attention as compared to recent growing recognition by global community. However, 29 (9%) and 3 (0.9%) health care providers replied that rabies do not attack dogs and humans, respectively. This implies that there could be less emphasis given to zoonotic disease in the curriculum or absence of ongoing refreshment training for health care providers in the district. This poor level of knowledge if left unresolved may be a significant obstacle for future prevention and control efforts on the disease.

More than 96% of the respondents mentioned that rabies can be transmitted from sick animal to human or vice versa. This is similar to study conducted among medical practitioners (94.3%) in Tanzania [15] whereas it is much higher than that conducted among Indian medical interns and residents (5%) [16]. This variation may be due to study participant difference and rabies epidemiology in the studied areas. Likewise, respondents mentioned that vaccination of animals (73.9%), isolation of sick animals (80.7%), creating community awareness (93.5%), and conducting active surveillance (87.6%) can prevent the acquisition and transmission of rabies in human and animal population. These are good entry points for introduction of zoonotic disease surveillance using One Health approach in the studied area.

Though the majority of the respondents heard about anthrax, significant number of them replied that anthrax do not attack human and animals. This implies that health care providers in the district can easily miss anthrax cases that can in turn contribute to a number of needless human and animal deaths and delayed outbreak detection and allow the disease to persist in the population. As a result, there could be difficulty in exporting animal products and the consequence is detrimental to the national economy. As high as fifty percent of the respondents did not know that anthrax can be transmitted from sick animal to human. Similarly half of the respondents did not know that anthrax can be prevented through vaccination, isolation of sick animals, creating community awareness, conducting active surveillance, wearing protective clothing, disinfection of contacts, and proper burial of dead animal/human from anthrax. This may be explained by the fact that, unlike other national priority infectious diseases, there are no national/international zoonotic disease prevention and control programs in the district. Such significant number of health care providers may not be able to advise on the method of anthrax transmission/acquisition prevention and this will lead to the persistent occurrence of anthrax outbreak in human and animal population.

The majority of the respondents replied that occurrence of rabies and anthrax in either population is reportable and both sectors can jointly conduct surveillance on the diseases. This implies that there is a fertile ground for establishment of joint surveillance system for zoonotic disease in the district.

Just over a third of health care providers reported that their respective sectors had conducted joint surveillance for zoonotic diseases of public health importance; nevertheless, their involvement was limited to joint outbreak response. In other words, the focus was on zoonotic disease outbreak control rather than zoonotic disease outbreak prevention. The need for joint response only during outbreaks of zoonotic disease in low income countries is to reduce the far reaching consequences of the outbreak. If such approach remains unresolved, there will be persistent occurrence of outbreaks, economic loses, and threatening lives of humans and animals. In addition, joint surveillance structure and manual at all levels were absent in both sectors. All of these issues imply a challenge for the realization of the new approach.

However, the finding for this study should be interpreted with the following limitations: health care providers from animal health sector were not adequately represented in our sample. Hence, the findings might not represent the knowledge and practice of these health care providers.

## 5. Conclusion

Almost all respondents replied that occurrence of both diseases in human and animals is reportable and the sectors can conduct joint surveillance on the two zoonotic diseases. A few respondents had past experience of joint zoonotic disease surveillance but it was only during outbreak response for such diseases. It was further revealed that there were no formal joint surveillance structure and its implementation manual in the district. Therefore, formal joint surveillance structure for rabies and anthrax should be in place for optimal implementation of all surveillance components.

## Figures and Tables

**Table 1:** Sociodemographic characteristics of health service providers, Gomma district, Jimma Zone, 2015.

Variable		Number (*n* = 323)	Percent (100%)
Sex	Male	111	34.4
	Female	212	65.6

Age in years	20–30	178	55.1
	31–40	123	38.1
	41–50	18	5.6
	51–58	4	1.2

Profession	B.S. nurse	48	14.9
	Diploma nurse	75	23.2
	Developmental agents	9	2.8
	Health officer	22	6.8
	Laboratory technologist	111	34.4
	Pharmacist	10	3.1
	Health extension workers	28	8.7
	Environmental Health	3	.9
	Animal science practitioners	17	5.3

Institution	Human district office	21	6.5
	Animal district office	7	2.2
	Health centre	239	74.0
	Health post	37	11.5
	Veterinary clinic	19	5.8

Marital status	Married	114	35.3
	Single	203	62.8
	Widowed	4	1.2
	Other	2	.60

Education level	Certificate	8	2.5
	Diploma	117	36.2
	B.S. degree	182	56.3
	Other	16	4.9

Religion	Orthodox	79	24.5
	Catholic	1	.3
	Protestant	141	43.7
	Islam	102	31.6

Ethnicity	Amhara	25	7.7
	Oromo	253	78.3
	Other	45	13.9

Work experience (in months)	1–12	36	11.1
	13–24	23	7.1
	25–36	30	9.3
	37–48	27	8.4
	49–60	36	11.1
	≥61	171	52.9

**Table 2:** Knowledge gap of health service providers on rabies and joint surveillance, Gomma district, Jimma Zone, 2015.

Variable			Number (*n* = 323)	Percent (100%)
Heard about rabies? (*n* = 323)		Yes	322	99.7
		No	1	.3

Rabies attack (*n* = 322)	Dogs	Yes	293	91.0
		No	27	8.4
		Not sure	2	.6
	Human	Yes	319	99.1
		No	3	.9
	Cats	Yes	162	50.3
		No	135	41.9
		Not sure	25	7.8
	Other animals	Yes	136	42.2
		No	182	56.5
		Not sure	4	1.2

Rabies transmission (*n* = 322)	Sick animal to human	Yes	316	98.1
		No	4	1.2
		Not sure	2	.6
	Sick animal to animal	Yes	311	96.6
		No	8	2.5
		Not sure	3	.9

Rabies prevention (*n* = 322)	Vaccination of animals	Yes	238	73.9
		No	73	22.7
		Not sure	11	3.4
	Isolation of sick animals	Yes	260	80.7
		No	58	18.0
		Not sure	4	1.2
	Creating community awareness	Yes	301	93.5
		No	16	5.0
		Not sure	5	1.6
	Conducting active surveillance	Yes	282	87.6
		No	28	8.7
		Not sure	12	3.7

**Table 3:** Knowledge gap of health service providers on anthrax, Gomma district, Jimma Zone, 2015.

Variable			Number (*n* = 323)	Percent (%)
Heard about anthrax? (*n* = 323)		Yes	315	97.5
		No	5	1.5
		Not sure	3	.9

Anthrax attacks (*n* = 315)	Domestic animals	Yes	211	67.0
		No	99	31.4
		Not sure	5	1.6
	Human	Yes	206	65.4
		No	106	33.7
		Not sure	3	1.0
	Wild animals	Yes	64	20.3
		No	221	70.2
		Not sure	30	9.5

Anthrax transmission (*n* = 315)	Sick animal to human	Yes	216	68.3
		No	95	30.2
		Not sure	6	1.6

Anthrax prevention (*n* = 315)	Vaccination of animals	Yes	202	64.1
		No	107	34.0
		Not sure	6	1.9
	Isolation of sick animals	Yes	198	62.9
		No	108	34.3
		Not sure	9	2.9
	Creating community awareness	Yes	205	65.1
		No	108	34.3
		Not sure	2	.6
	Conducting active surveillance	Yes	175	55.6
		No	131	41.6
		Not sure	9	2.9
	Wearing protective clothing	Yes	175	55.6
		No	130	41.3
		Not sure	10	3.2
	Disinfection of contacts/materials	Yes	196	62.2
		No	116	36.8
		Not sure	3	1.0
	Properly burial of dead animal/human	Yes	199	63.2
		No	112	35.6
		Not sure	4	1.3

Are human rabies and/or anthrax reportable diseases in Ethiopia?		Yes	316	97.8
		No	2	.6
		Not sure	5	1.5

Are animal rabies and/or anthrax reportable diseases in Ethiopia?		Yes	310	96.0
		No	6	1.9
		Not sure	7	2.2

Do you think that human and animal sectors can	Work together for zoonotic diseases	Yes	316	97.8
		No	5	1.5
		Not sure	2	.6
	Jointly conduct surveillance on zoonotic diseases	Yes	313	96.9
		No	6	1.9
		Not sure	4	1.2

**Table 4:** Practice gap of health service providers and their respective sectors on joint surveillance, Gomma district, Jimma Zone, 2015.

Variable			Number (*n* = 323)	Percent (100%)
Conducted joint surveillance on zoonotic diseases with human or animal sector		Yes	117	36.2
		No	194	60.1
		Not sure	12	3.7

Joint surveillance activity experience (*n* = 117)	Planning	Yes	11	9.4
		No	106	90.6
	Surveillance data exchange	Yes	13	11.1
		No	103	88.0
		Not sure	1	.9
	Outbreak response	Yes	116	99.1
		No	1	.9
	Monitoring and evaluation	Yes	12	10.3
		No	105	89.7

Did your sector have integrated surveillance structure?		Yes	7	2.2
		No	299	92.6
		Not sure	17	5.2

Level of integration was between (*n* = 7)	Health extension workers and DAs	Yes	5	71.4
		No	2	28.6
	Health centre and veterinary clinic	Yes	5	71.4
		No	2	28.6
	Human and animal focal persons	Yes	5	71.4
		No	2	28.6

Integrated surveillance manual in your institution		Yes	6	1.9
		No	302	93.5
		Not sure	15	4.6
